# Aspirin and Infection: A Narrative Review

**DOI:** 10.3390/biomedicines10020263

**Published:** 2022-01-25

**Authors:** Stefano Di Bella, Roberto Luzzati, Luigi Principe, Verena Zerbato, Elisa Meroni, Mauro Giuffrè, Lory Saveria Crocè, Marco Merlo, Maria Perotto, Elisabetta Dolso, Cristina Maurel, Antonio Lovecchio, Eugenia Dal Bo, Cristina Lagatolla, Bruna Marini, Rudy Ippodrino, Gianfranco Sanson

**Affiliations:** 1 Clinical Department of Medical, Surgical and Health Sciences, University of Trieste, 34127 Trieste, Italy; stefano932@gmail.com (S.D.B.); roberto.luzzati@asugi.sanita.fvg.it (R.L.); lcroce@units.it (L.S.C.); marco.merlo@asugi.sanita.fvg.it (M.M.); maria.perotto@asugi.sanita.fvg.it (M.P.); gsanson@units.it (G.S.); 2 Clinical Pathology and Microbiology Unit, “S. Giovanni di Dio” Hospital, 88900 Crotone, Italy; luigi.principe@gmail.com; 3 Infectious Diseases Unit, Trieste University Hospital, 34149 Trieste, Italy; verena.zerbato@asugi.sanita.fvg.it (V.Z.); elisabetta.dolso@gmail.com (E.D.); cristina.maurel@asugi.sanita.fvg.it (C.M.); antonio.lovecchio@asugi.sanita.fvg.it (A.L.); 4 Clinical Microbiology and Virology Unit, “A. Manzoni” Hospital, 23900 Lecco, Italy; el.meroni@asst-lecco.it; 5 Cardiothoracic-Vascular Department, Azienda Sanitaria Universitaria Integrata, Cattinara University Hospital, 34149 Trieste, Italy; eugenia.dalbo@asugi.sanita.fvg.it; 6 Department of Life Sciences, University of Trieste, 34127 Trieste, Italy; clagatolla@units.it; 7 Ulisse BioMed Labs, Area Science Park, 34149 Trieste, Italy; b.marini@ulissebiomed.com (B.M.); ippodrino@gmail.com (R.I.)

**Keywords:** aspirin, acetylsalicylic acid, ASA, infection, infections, infectious disease, infectious diseases, biofilm, anti-biofilm, virus

## Abstract

Acetylsalicylic acid (ASA) is one of the most commonly used drugs in the world. It derives from the extract of white willow bark, whose therapeutic potential was known in Egypt since 1534 BC. ASA’s pharmacological effects are historically considered secondary to its anti-inflammatory, platelet-inhibiting properties; however, human studies demonstrating a pro-inflammatory effect of ASA exist. It is likely that we are aware of only part of ASA’s mechanisms of action; moreover, the clinical effect is largely dependent on dosages. During the past few decades, evidence of the anti-infective properties of ASA has emerged. We performed a review of such research in order to provide a comprehensive overview of ASA and viral, bacterial, fungal and parasitic infections, as well as ASA’s antibiofilm properties.

## 1. Introduction

Aspirin (acetylsalicylic acid) is one of the most commonly used drugs in the world [1]. It derives from the extract of white willow (*Salix alba*) bark. The first evidence of the therapeutic use of *salix* (now known as willow) dates back to 1534 BC from the Egyptian Ebers Papyrus, considered the most comprehensive medical papyrus ever recovered [2]. Then, the use of willow bark continued through ancient Greece and through to Roman times [1]. In the 19th century, scientists were able to produce a compound from the crystals isolated from willow bark which was named salicylic acid [3] and in 1852, salicylic acid was acetylated for the first time to reduce its irritant properties. In 1899, aspirin was coined.

After more than 100 years, acetylsalicylic acid (ASA) is still a first-line drug for several clinical needs. The application is mainly as an analgesic, antipyretic and antiplatelet drug. However, interesting properties as anticancer agents have been demonstrated, particularly for colorectal cancer [4].

Nowadays, there is a significant amount of data on the relationship between ASA and infectious diseases. Indeed, ASA has properties affecting the immune response, as well as properties with intrinsic anti-infective and anti-biofilm activity.

**Figure 1:**
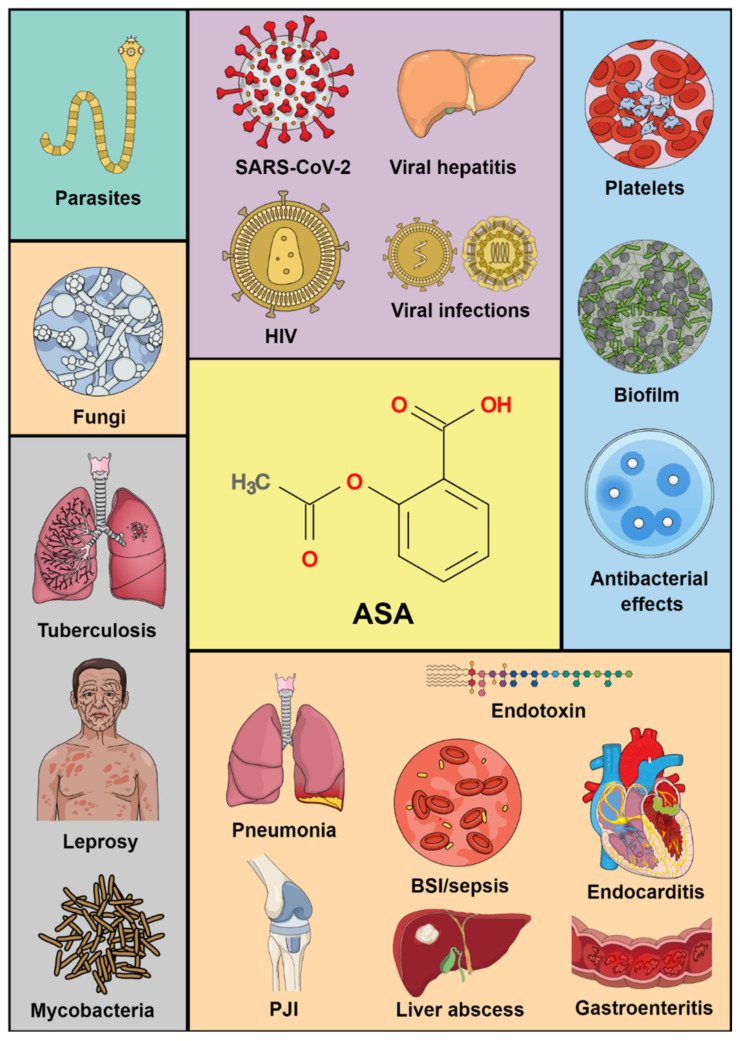
Potential targets of acetylsalicylic acid in infectious diseases. ASA: acetylsalicylic acid; BSI: bloodstream infection; HIV: human immunodeficiency virus; PJI: prosthetic joint infections.

There are several lines of evidence on ASA’s effects on bacteria, viruses, fungi, parasites and their associated infections (Figure 1). Therefore, the aim of this study was to review the literature on the association between ASA and infectious diseases.

## 2. Methods

We searched articles in PubMed and Scopus databases using the following as a common search string: (“aspirin” OR “ASA” OR “acetylsalicylic acid” OR “acetylsalicylate”). For each section of interest, this string was expanded with appropriate search terms linked with the Boolean operator AND (e.g., COVID-19 or SARS-CoV-2). The last search was performed on 17 November 2021. Results were filtered to automatically exclude studies published in languages other than English.

From these search results, we selected papers focused on systemic diseases, while studies related to *Helicobacter pylori* infection, rhinitis, sinusitis, rhinosinusitis, gingivitis and periodontitis were excluded. On this basis, we reviewed articles judged more relevant, aiming to provide a comprehensive overview based on available literature related to the role of ASA’s effects on infectious diseases. The results are presented in separate sections consistent with the searched literature, where the findings are presented and briefly discussed. The most relevant results of the review are summarized in Table 1.

## 3. Results

### 3.1. Aspirin, Platelets and Infections

ASA anti-inflammatory and anti-platelet effects are largely dose-dependent. Low doses have mainly antiplatelet effects, while high doses have mainly anti-inflammatory effects. All considered studies focused on bacterial organisms. Moreover, available studies investigated ASA’s effects on platelets after exposure to a restricted number of bacteria (*Staphylococcus aureus*, *Enterococcus faecalis*, *Streptococcus sanguinis*, *Escherichia coli*) [5,6].

Chabert et al. [6] conducted a study on the effects of ASA on induced thrombocytopenia in human platelets exposed to *S. aureus* strains. Freshly obtained platelets were exposed to different *S. aureus* strains for 30 min and the investigators observed expressed activation markers, a significant decrease in platelet count, the expression of cell death markers and the release of RANTES and GROα. In addition, none of the tested strains induced platelet aggregation, except for 5 out of 35 donors. This peculiar phenomenon is interpreted as a host specificity of the response. Subsequently, they assessed the ability of ASA to prevent the platelet decrease after exposure to clinical *S. aureus* strains. According to their findings, in vitro ASA (500 µM) limited platelet activation and inflammatory factor release and restored the platelet count by protecting platelets from *Staphylococcus*-induced expression of cell death markers [6].

Similar in some aspects, but not in the results, Hannachi et al. [5] investigated the distinct effects of ASA on platelet aggregation induced by infectious bacteria. This work demonstrated that different bacterial strains induced different kinds of platelet aggregates. The bacterial species tested were *S. aureus*, *E. faecalis* and *S. sanguinis*. Furthermore, they found that ASA was able to reduce bacteria-induced platelet aggregation depending on the bacterial species. They used “control platelets” from healthy subjects’ blood (n = 17) vs. platelets from blood of patients under daily ASA therapy at 75–160 mg/day (n = 15). The experiments in vitro showed that ASA significantly reduced platelet aggregation induced by *S. aureus* (*p* = 0.003) and *E. faecalis* (*p* = 0.006), but no effects were seen in the case of *S. sanguinis* (*p* = 0.529) [5]. The point in favor of this study is the testing of platelet-activation effects under the stimulus of different bacterial species, showing that there are significant differences. This perhaps overcame discordant results of previous studies where differences in bacterial species were not considered [7,8,9].

Controversies about this topic are evident and underlined in many works. The difficulty in comprehension comes from the complexity of the multiple mechanisms involved. Both bacteria (e.g., *S. aureus*) and platelets express a number of receptors according to the stimuli they receive.

It is demonstrated that platelets reserve many receptors which, when triggered, may [10,11] or may not [12,13] lead to platelet aggregation. Other works, including both studies described here, showed that platelets can distinguish inflammatory from aggregation responses depending on the bacterial types [14,15] or concentration [16].

In brief, the available data confirm that platelet secretion triggered by infectious pathogens is not subjected to an “all or none” mechanism; consequently, platelet inhibitors, such as ASA, also have differential and elaborate effects on these secretion patterns. In fact, the use of ASA during bacterial infections at different concentrations can lead to different effects on platelets. As an example, the administration of low doses of ASA [6,7,8,9] showed the ability to modify platelet functions with protective effects. Conversely, ASA used at 5 mM (10 times higher than in Chabert’s study) induced platelet apoptosis via the upregulation of pro-apoptotic proteins or mitochondrial pathways [17,18].

In conclusion, ASA, having direct and indirect effects on platelets, may be a promising molecule to limit the severity of sepsis, but more in-depth and structured studies are needed.

### 3.2. Aspirin and Antibiofilm Effects

Biofilms (BF) are complex structures formed by aggregates of microorganisms growing in an extracellular matrix, which creates a protective environment against the immune response and antimicrobials. The sessile cells inside BF are highly resistant to antimicrobials; therefore, a variety of non-antibiotic pharmacological strategies have been investigated. Among them, ASA looks promising, especially against fungi.

Alem and Douglas showed that a 48 h treatment of *Candida albicans* grown on polystyrene with 200 µM ASA inhibited 80% of the sessile cells [19], and in a more recent study, 40 mg/mL ASA was able to eradicate different *Candida* species BF on silicone. In detail, *C. albicans* showed the highest susceptibility and was completely eradicated after only 4 h of treatment, while other species required 24 h to achieve the same result [20]. A lower but still present activity was also observed against bacterial BFs, as a 1.5 h treatment caused a 30% removal of the BF formed by *Staphylococcus epidermidis* and *P. aeruginosa* on polystyrene [21].

The ability of ASA to enhance the activity of various antimicrobials against BF have also been investigated. ASA tested in combination with caspofungin on 39 clinical isolates of *C. albicans* showed mostly synergistic (41%) or additional (54%) activity and restored drug susceptibility in 41% of the isolates [22]. Favorable synergism was also detected against *Trichosporon ashii* with amphotericin B (67%), caspofungin and fluconazole (53%), while lower activity was observed in combination with itraconazole (33%) and voriconazole (13%) [23].

These anti-BF properties of ASA seem promising in terms of its potential use as an alternative drug for the catheter-lock technique [24].

ASA has also been investigated for its ability to inhibit BF formation, in this case with controversial results. In fungi, the addition of ASA <1 mM to the culture medium resulted in a 50% decrease in BF production by six different *Candida* species [25] and in another study, the same result was obtained for *C. albicans* even at 100 µM ASA, a concentration normally achieved in the serum after ingestion of ASA at doses within the therapeutic range [19]. This anti-BF activity was related to the inhibition of cyclooxygenase, an enzyme involved in the biosynthesis of prostaglandins, which are important for the development of *C. albicans* BF [19].

Different results were obtained when testing bacteria. The production of BF was reduced by 50%, but only at high concentrations of ASA: 5 mM for *Staphylococcus epidermidis* [26] and 32 mM for *P. aeruginosa* [27]. Moreover, when *S. aureus* strains were tested, no inhibition was observed against an MRSA incubated in ASA 10 mM, and an increase in BF production was even observed in a laboratory strain grown in a medium containing ASA 0.3–2 mM [28].

Most available data on the anti-BF activity of ASA come from in vitro studies and their results are often difficult to compare because different techniques and different ASA concentrations were used. However, the main limitation arising is that inhibitory activity is mostly detected at suprapharmacological concentrations of the drug. Nevertheless, an in vivo study performed on an animal model showed that streptococcal adhesion to injured heart valves was significantly reduced when bacterial inoculation was preceded by ASA administration [29]. In addition, an interesting in vivo study was recently conducted to investigate the effects of ASA administration on the resolution of periprosthetic joint infection (PJI). Follow-up of 88 patients over a 9-year period showed that patients who had received regular ASA for >6 months prior the diagnosis of PJI, and who took the drug ≥100 mg/day during the PJI treatment course, showed a better resolution of the infection [30]. In conclusion, even if the anti-BF activity of ASA in vitro is detected at suprapharmacological concentrations, the few available in vivo studies suggest a beneficial effect of this drug against BF-related infections. Further in vivo studies are required to confirm this hypothesis.

### 3.3. Aspirin and Bacterial Infections

#### 3.3.1. Antibacterial General Effects

ASA is rapidly metabolized to salicylic acid. In addition to its anti-inflammatory effects, salicylic acid could also inhibit bacterial growth, showing antibacterial effects. The antibiotic properties of ASA have been investigated in the past 2 decades, with several studies reporting on the activity of ASA at different levels.

Bartzatt et al. explored the pharmacodynamics of non-steroidal anti-inflammatory drugs (NSAIDs) and the activities of tripeptide substituents [31]. The structures of tripeptide consisted of a reactive site for antibacterial activity and a linker to the NSAID carrier drug. NSAIDs served as carrier drugs of the N-mustard group, which expressed alkylation activity. The results demonstrated that the ASA N-mustard agent expressed strong antibacterial activity against *E. coli*. In 1971, Jones et al. [32] studied the effect of ASA on infections in children with rheumatic reaction. The results showed that the level of salicylate inhibited the growth of some of the tested microorganisms. ASA showed bacteriostatic action against Gram-positive bacteria, but no activity against Gram-negative bacteria [33]. In another paper, Jones et al. confirmed that ASA has considerable toxicity for Gram-positive cocci and less for Gram-negative rods [32]. Hockert et al. analyzed the in vivo influence of ASA on *Listeria monocytogenes* infection in spleen and liver of mice. ASA was able to reduce bacterial burden in mice infected with *L. monocytogenes*. Specifically, the prophylactic administration of ASA in a concentration of 5 mg/kg body weight resulted in a more than 10-fold reduction in *L. monocytogenes* [34]. Yang et al. [35] analyzed a retrospective study of Sedlacek et al. [36] which stated that a high dose of ASA (324 mg) rather than a low dose (81 mg) was associated with a decreased risk of *S. aureus* infection in hemodialysis patients. However, high doses of ASA cause a depletion of iron stores that are critical for bacterial metabolism. Chung et al. (1998) evaluated the effects of ASA on arylamine N-acetyltransferase (NAT) activities in *Klebsiella pneumoniae*. The enzyme catalyzes the transfer of acetyl groups from acetyl-CoA to arylamines and it is involved in several metabolic functions (i.e., inactivation of drugs). The data indicate decreased NAT activity associated with increased levels of ASA in *K. pneumoniae* cytosol [37].

Effects of ASA were also evaluated against *Chlamydophyla pneumoniae*, a Gram-negative obligate intracellular bacterium. Tiran et al., in 2002, demonstrated that ASA counteracts the intracellular development and maturation of chlamydial inclusions [38]. The following year, Yoneda et al. confirmed the effect of ASA on chlamydial growth through the activation pathways of NFkB, COX-2 and prostaglandin E2. The results indicated that ASA had anti-inflammatory activity through the prevention of NF-kB activation and anti-chlamydial activity at high doses [39].

Interestingly, ASA can influence bacterial susceptibility to antibiotics. Zimmermann et al. (2018) claimed that salicylic acid co-administered during treatment of infections has significant effects on bacteria that frequently can compromise the effectiveness of antibiotics, by increasing/decreasing outer-membrane proteins, efflux pumps and up-regulating antibiotic targets [40]. Malla et al. (2020) investigated chemical molecules able to convert colistin resistance [41]. Recently, the conversion of colistin resistance induced by carbonyl cyanide-m-chlorophenylhydrazone in *E. coli* and *K. pneumoniae* has been described. The results found that ASA partially or totally reverted resistance to colistin.

The impact of ASA on the bacterial quorum sensing (QS) system has also been evaluated [27]. In particular, the study of El-Mowafy et al. (2014) suggested the QS inhibitory action of ASA in *Pseudomonas aeruginosa*, with reduced production of elastase, proteases and pyocyanin. ASA also provided a reduction in bacterial motility [27].

Furthermore, ASA showed antibacterial properties when combined with other molecules. Ngaini et al. (2019) analyzed the synthesis of aspirinate-halogenated azo, halogenated azo and azoaspirin derivatives. Halogenated azo-aspirin derivatives exhibited better antibacterial activities against both Gram-negative and Gram-positive bacteria than the ampicillin and ASA alone [42]. Fajstavr et al. (2020) demonstrated the use of a nanostructured honeycomb-like pattern doped with ASA [43]. Selected nanostructured surfaces were confirmed to have excellent antibacterial properties when tested against a model bacterial strain of *S. aureus* [43].

Overall, these data highlight a promising antimicrobial application of ASA.

#### 3.3.2. Aspirin and Endotoxin (Gram-Negative Lipopolysaccharide)

Several studies investigated the potential of ASA in mitigating lipopolysaccharide (LPS)-related harmful effects. The idea behind them was to use ASA in septic patients, it being known that endotoxic shock is associated with increased metabolism of arachidonic acid into thromboxanes and prostaglandins. The most available studies were dated, conducted on animal models and evaluated heterogeneous outcomes.

Studies conducted in the 1970s and 1980s in rats and rabbits were promising, often demonstrating a significant decrease in mortality when animals exposed to LPS were pre-treated with ASA [44,45,46]. A dated study conducted on primates (baboons) demonstrated that ASA (40 mg/kg of body weight) could prevent the precipitate falls in the renal artery flow (*p* < 0.01) and platelet count (*p* < 0.01), which occur 3 min after the intravenous injection of endotoxin (7 mg/kg) [47]. From the 1990s, human studies began to be published: in 1999, a study on 30 healthy young men who received 4 ng/kg LPS showed no effect of ASA pretreatment (1 g) on LPS-induced changes in adhesion parameters; however, the ASA dose administered was far from reaching the IC_50_ [48]. A decade later, a study on 10 healthy male volunteers demonstrated that ASA (425 mg every 12 h for 7 days) attenuated the endotoxin-induced platelet plug formation and decreased the urine 11-dehydro-thromboxane B2 levels, while no effects on soluble P-selectin or VWF-levels were documented [49]. In 2017, another study randomized healthy volunteers to receive a placebo or ASA 75 or 1200 mg for 7 days prior to LPS inhalation (50 μg). ASA did not reduce bronchoalveolar IL-8, but it reduced pulmonary neutrophilia and tissue-damaging neutrophil proteases (matrix metalloproteinase (MMP)-8/-9), reduced bronchoalveolar concentrations of tumor necrosis factor α and reduced systemic and pulmonary thromboxane B2. There was no difference between high-dose and low-dose ASA [50]. The same year, a study on 40 healthy subjects demonstrated that ASA pretreatment profoundly augmented the pro-inflammatory cytokine response after LPS challenging (1 ng/kg followed by 1 ng/kg/h during 3 h) [51]. Recently, a double-blind, randomized, placebo-controlled study in healthy volunteers confirmed this. Volunteers received endotoxin twice (1 ng/kg followed by 1 ng/kg/h during 3 h) at 1-week intervals and were randomized to ASA prophylaxis (80 mg daily for 14 days, starting 7 days before the first endotoxin challenge), ASA treatment (80 mg daily for the 7-day period between both endotoxin challenges) or placebo. The results showed that ASA treatment induced a proinflammatory phenotype with enhanced plasma levels of tumor necrosis factor-α (+53%; *p* = 0.02), IL-6 (+91%; *p* = 0.03), and IL-8 (+42%; *p* = 0.02), whereas plasma levels of the key anti-inflammatory cytokine interleukin-10 were attenuated (−40%; *p* = 0.003). The authors concluded by proposing ASA for sepsis-induced immunoparalysis [52].

#### 3.3.3. Aspirin and Bloodstream Infections/Sepsis

The topic “aspirin and sepsis” has seduced many scientists. The interest in this topic also rose as a result of encouraging data from animal studies conducted in the 1970s [53]. The rationale lies in the awareness that platelets, interacting with endothelial cells and leukocytes, might promote excessive systemic inflammation and hypercoagulopathy; therefore, an antiplatelet drug such as ASA could mitigate these effects.

A recent well-conducted experimental study of *S. aureus*-induced sepsis in mice showed that animals pretreated with ASA or animals receiving ASA 3 h post-infection had significantly reduced platelet aggregation and neutrophil extracellular trap release. Additionally, ASA-treated mice had reduced intravascular thrombin activity and microvascular occlusion [54].

However, when looking at human studies, results are less encouraging. In 2016, a propensity score-matched retrospective study analyzed 836 and 602 episodes of *S. aureus* and *Escherichia coli* bloodstream infection, respectively. Low-dose ASA was associated with a reduced short-term mortality in patients with *S. aureus* (12% vs. 27% 30-day all-cause mortality; 14% vs. 27% in-hospital all-cause mortality), but not *E. coli* bloodstream infection [55]. In 2017, an individual patient data meta-analysis of observational studies describing the association between ASA use prior to the onset of sepsis and mortality in hospitalized patients was published. A total of 6823 records were used for analysis and the two-step random effects meta-analysis showed a pooled estimate of a significant reduction in risk of death by 7% (95% CI, 2–12%; *p* = 0.005), with significant statistical heterogeneity (I^2^ = 61.6%) [56]. In 2018, data from 29,690 adults ≥45 years old were analyzed to evaluate the association between ASA use and sepsis hospitalization. Forty-three percent of individuals reported ASA use and the median follow-up was 6.2 years. No associations between long-term exposure to ASA and onset of sepsis and sepsis-related death were found [57]. In 2020, the ANTISEPSIS trial was published: 16,703 participants aged 70 years and older without major illnesses were enrolled and followed up for a median of 4.6 years. The study was a community-based, randomized, placebo-controlled, double-blind trial of low-dose ASA (100 mg per day) for the primary prevention of sepsis: 8322 participants received ASA and 8381 received placebo, and 203 deaths were considered to be associated with sepsis. No differences between the two arms were found [58].

These results may not be conclusive. In fact, it is known that platelet activation is significantly different according to the bacterium in question and *S. aureus* is a strong platelet “activator”, but primary endpoint events (deaths) due to *S. aureus* were only five in the ANTISEPSIS trial and this should be taken into account when interpreting the results.

#### 3.3.4. Aspirin and Endocarditis

Infective endocarditis (IE) is an infection of the endocardium, mostly of bacterial origin. Its cornerstone lesion is the vegetation, a mass of platelets and fibrin forming in response to endothelial injury, onto which bacterial colonization develops during bacteremia. Platelet microbial-induced activation and adhesion to damaged endocardium is a pathophysiological *primum movens*. As such, studies over the years have sought to determine whether ASA, with its known antiplatelet properties, could limit vegetation formation and, consequently, its embolization, a dreadful consequence of IE possibly leading to stroke in up to 30% of patients.

In 1977, ASA was tested on an animal model of *Streptococcus viridians* endocarditis. Rabbits were pre-treated with ASA (600 mg) before catheter-induced injury of the aortic valve and bacterial inoculation. Effects on early thrombus formation were assessed by vegetation weight, size, and number of bacterial colonies. No difference was found in thrombus formation nor subsequent IE development between groups [59].

Fifteen years later, Nicolau et al. used a similar model (rabbit) and approach to test a lower dose of ASA (5 mg/kg) in *S. aureus* IE, after valve injury. They found a non-significant 30% reduction in vegetation weight with a significantly reduced bacterial density [60]. The first human study was contemporary: an observational prospective study on nine patients with native or prosthetic valve IE, randomized to low-dose ASA (75 mg/day) or none. Stroke incidence and vegetation area by echocardiography were assessed over a two-year period. In that experience, a lower incidence of embolic events (2 controls, 0 treated) along with a decreased vegetation area was suggested [61].

Animal experiments that came afterwards hypothesized a dose-dependent ASA effect, finding that 8–10 mg/kg significantly sterilize vegetations and halt their growth, and ruling out its direct antimicrobial activity by MIC testing [62,63].

The aforementioned studies prompted a randomized double-blind placebo-controlled trial enrolling a small population (115 patients vs. 368 planned) with native and prosthetic valve IE and testing 325 mg/day ASA against a placebo for 4 weeks [8]. No difference in vegetation size nor valvular dysfunction was found, along with a non-significant trend towards more embolic events in the treated group (28% vs. 20%), in which the risk for bleeding was nearly two times higher (*p* = 0.07).

Finally, a meta-analysis of human studies without confounders assessment demonstrated a significantly reduced embolism, no differences in bleeding but a trend towards higher mortality in patients treated with ASA in IE [64]. These results discouraged further investigations, deeming the possible benefits of ASA pretreatment insufficient to warrant its use. Current literature speculates on a possible aspirin-enriched antibiotic prophylaxis before at-risk procedures in susceptible populations [65,66]. Nevertheless, definite data are lacking.

In conclusion, only a few studies on IE treatment with ASA have been published, mostly on animals. The majority of studies are retrospective and enrolled small populations, with heterogeneous time of ASA administration, its dosage and the infectious species involved. The results are controversial, but a clear benefit of ASA treatment in IE has not emerged. Therefore, to date, guidelines for IE management do not recommend ASA.

#### 3.3.5. Aspirin and Gastroenteritis/Liver Abscess

Since the late 1970s, there has been interest in the efficacy of antisecretory drugs in secretory diarrhea (e.g., cholera). ASA was reported to have antisecretory properties by blocking endogenous prostaglandin synthesis [67] or by interfering with the secretory process, as shown by experiments in normal rabbit ileum (stimulating Na^+^ and Cl^−^ reabsorption) [68] or cat intestine (inhibiting cholera toxin-induced secretion) [69]. However, it appears that ASA prevents the action of cholera toxin if administered before its net absorption by intestinal cells, but it cannot reverse the toxin action [70].

Findings in human studies are conflicting, with evident differences between children and adults. It is also worth noting that the below-mentioned studies did not perform subgroup analyses, especially in terms of etiological factors. Burke et al., in a double-blind trial where they administered 300 mg of ASA (or placebo) orally to moderately/severely dehydrated children with acute gastroenteritis, reported that stool volume decreased significantly in ASA-treated individuals, with a mean difference of 100 mL (*p* < 0.05) [71]. Moreover, the children treated with ASA showed a two-fold mean weight gain compared to the other two groups.

Similar results were reported by Gracey et al., who randomized 68 children with acute diarrhea to treatment (Aspro Clear™ tablets, containing 300 mg of ASA, 606 mg of sodium bicarbonate, and 400 mg of anhydrous citric acid) or placebo. Patients in the treatment group showed a shorter duration of diarrhea (3.83 vs. 2.72 days, *p* < 0.05), decrease in stool volume (7.37 mL vs. 4.66 mL per kg body weight per day, *p* < 0.05) and increase in body weight gain (80.6 g/day vs. 59 g/day, *p* < 0.05) [72]. In another study, 70 adults admitted with acute diarrhea were randomized into a treatment group (n = 33), who received 1500 mg of ASA per day orally, and a placebo group (n = 37). The results showed no significant difference in terms of symptom regression and overall diarrhea duration [73]. Similar data were also reported by Islam et al., who demonstrated that ASA in a dose of 25 mg/kg/day given in four equally divided doses did not reduce the stool output and disease duration [74].

ASA’s role has been also assessed in pyogenic liver abscesses (PLA). It has been reported that ASA can reduce capsule polysaccharide production by up to 70% [75], which resulted in the primary mechanism behind *K. pneumoniae* serotype K1′s increased susceptibility to leukocyte phagocytosis, preventing more invasive infections [76]. Liu et al. identified 5912 patients with PLA and evaluated recurrence PLA rates between ASA users and non-users, which were 42.5 and 74.6 per 1000 person-years of follow-up, respectively [77]. Similar findings were reported in a large cohort of diabetic patients, with ASA continuous intake for >90 days associated with a lower risk of PLA [78].

#### 3.3.6. Aspirin and Prosthetic Joint Infections

A potential role of ASA in reducing the risk of prosthetic joint infections (PJI) has been investigated. In 2015, the efficacy and safety of ASA (325 mg bis in die) in preventing venous thromboembolism (VTE) following total joint arthroplasty was investigated. According to this study, the incidence of PJI was significantly lower in patients receiving ASA (1456 patients) vs. those receiving warfarin (1700 patients) (0.4% vs. 1.5%, *p* < 0.001). However, this study had an important bias: patients determined to be at high risk for VTE by the treating surgeon (it could be speculated the more comorbid patients) did not receive ASA [79].

In 2019, using a mouse model of orthopedic implant-associated infections (*S. aureus*), researchers demonstrated that treatment with moderate doses of ASA resulted in decreased periosteal reactive bone, osteolysis and osteoclast activation but increased osteoblast activation in implant-associated infections mice, whereas a high dose of ASA aggravated rather than alleviated implant-associated infections [80].

In 2020, in a human clinical research on this topic, PJI patients with regular exposure to ASA (>6 months at ≥100 mg/day) were compared to patients not receiving ASA. Patients with PJI and ASA exposure had a benefit on infection resolution (multivariate analysis: HR 2.2; CI 1.018–4.757; *p* = 0.045) [30], but the authors did not find a higher treatment success rate in the ASA group compared to previous studies.

The question remains open.

#### 3.3.7. Aspirin and Pneumonia

The beneficial effects of ASA against the mechanisms protracting pulmonary damage in pneumonia are quite well recognized.

Early in 1976, Eyre et al. observed a clinical and histopathological improvement in ASA-treated calves with acute interstitial pneumonia caused by *Ascaris suum* [81].

Two relatively old studies suggested a possible role of ASA in improving arterial oxygenation by altering the regional distribution of pulmonary blood flow. Light measured a large reduction in intrapulmonary shunt from a mean baseline value of venous admixture-like perfusion (Qva/Qt) of 0.35 ± 0.05 to 0.25 ± 0.06 (*p* < 0.05) and a significant rise in PaO_2_ from 51 ± 8 mmHg to 62 ± 5 mmHg (*p* < 0.05) 60 min after intravenous administration of ASA 100 mg/kg in an animal lobar pneumococcal pneumonia model [82]. Conversely, a small human study failed to demonstrate an improvement in arterial oxygenation after the infusion of 2 g ASA in seven mechanically ventilated patients with unilateral pneumonia [83].

Another important ASA action is the modification of COX-2 action favoring the production of specialized pro-resolving mediators. Wang et al. evaluated the promotion of tissue inflammation resolution after the administration of aspirin-triggered Resolvin D1 (AT-RvD1) in a murine model of *Streptococcus pneumoniae* and influenza A virus coinfection. A reduction of approximately 50% in infiltrating monocytes/macrophages numbers, 30% in myeloperoxidase activity and approximately 13% in neutrophil elastase activity was demonstrated. Concurrently, bronchoalveolar lavage fluid (BAL) from AT-RvD1-treated mice showed a 20% (*p* < 0.05) inhibition of pneumococcal growth [84]. In Abdulnour’s study, AT-RvD1 enhanced macrophage phagocytosis of *E. coli* and *P. aeruginosa*, decreased BAL LPS, increased BAL lipocalin-2 and accelerated neutrophil efferocytosis to prevent excessive lung injury [85]. Excessive impairment of the normal pulmonary inflammatory response has been observed in mice with pneumococcal pneumonia treated with a high dose of ASA (4 to 6 g/day) [86].

The bidirectional relationship between cardiovascular (CV) diseases and lower respiratory tract infections is of growing interest. ASA could be a promising candidate for the prevention of CV events after pneumonia [87]. Falcone et al. retrospectively evaluated 1005 hospitalized patients with community-acquired pneumonia (CAP) and reported 8.3% non-fatal CV events within 30 days among ASA non-users versus 4.9% among chronic ASA users. In the latter group, the hospital death hazard ratio was reduced to 0.43 (95% CI 0.25–0.75) [88]. Additionally, a small, open-label Turkish randomized trial reported a reduction in CV events after CAP from 10/94 (11%) in the control group to 1/91 (1%) in patients treated with ASA 300 mg/day for 1 month [89]. Recently, Hamilton et al. performed a prior event rate ratio analysis with propensity score matching in a large primary care database and demonstrated a significant reduction in CV events up to 6 months after pneumonia in ASA users (493 vs. 877 events) and a significant association of prior ASA use with reduced myocardial infarction and stroke (HR 0.64, 95% CI 0.52–0.79) [90]. On the other hand, a large retrospective cohort study in Taiwan showed the association between long-term, low-dose ASA use and decreased risk of pneumonia in patients with cardio- and cerebrovascular ischemic diseases (adjusted HR 0.890, CI 0.837–0.945) [91].

It has been hypothesized that a combination of low-dose ASA and macrolide could be beneficial in severe acute pneumonia. Indeed, in a small prospective observational study on patients with septic shock from CAP, receipt of chronic ASA therapy plus a macrolide was associated with a reduced risk of 30-day death (HR 0.24, 95% CI 0.08–0.79, *p* = 0.01) [92]. A protective effect of this combination was then demonstrated in a larger cohort of patients with severe CAP. Despite the study limitations, the ASA plus macrolides group was associated with the lowest mortality rate (15.5%) and with a reduced risk of 30-day death (HR 0.71 95% CI 0.58–0.88 *p* = 0.002) after propensity score weighting [93].

#### 3.3.8. Aspirin and Mycobacterial Diseases

ASA is known to act against *Mycobacterium tuberculosis* by aspirin-triggered lipoxins that exert anti-inflammatory effects, reducing the burden of granulomas and the likeliness of death. ASA may also reduce *M. tuberculosis* growth, through the inhibition of tumor necrosis factor α via NF-κB stabilization. Tuberculous meningitis (TBM) is the most severe form of tuberculosis, characterized by inflammation and arteritis of the small- and medium-sized intracranial vessels, resulting in stroke and other neurological sequelae. The role of corticosteroids in TBM is now established, while some early-phase clinical trials recently evaluated the efficacy and safety of ASA in patients with TBM. In a randomized open-label trial, patients with TBM were treated with anti-tuberculosis therapy (ATT) and steroid therapy, plus either placebo or low-dose ASA (150 mg). The addition of ASA resulted in significantly lower mortality compared to the placebo group (21.7% and 43.4%, respectively, *p* = 0.02) [94]. A more recent study addressed the possible benefit of ASA plus corticosteroids alongside ATT for TBM. A total of 135 patients were included and treated as follows: group (1) received ASA alone plus ATT, group (2) received both ASA and steroids plus ATT and group (3) received ATT alone. At 3 months, 31 (23%) patients died; no statistically significant difference in mortality between groups was found [95].

Finally, a systematic review of the role of ASA in TBM [96] confirmed that it reduces the risk of new cerebral infarction, but it does not affect mortality.

Regarding non-meningeal tuberculosis, in recent years, one study provided information on different ASA dosages and their effects in a murine tuberculosis model, confirming that low-dose ASA significantly increased survival, even enhancing the effect of ATT [97]. More recently, ASA was investigated in a randomized controlled trial in which patients with pulmonary tuberculosis and type 2 diabetes were recruited and randomized to intervention (100 mg of ASA per day) versus placebo: ASA provided clinical benefits in terms of improved clinical signs and symptoms and cavitary lesions, reduced inflammatory markers and higher rates of sputum-negative conversion [98].

Leprosy is a spectrum of diseases caused by *Mycobacterium leprae*, and it is strongly dependent on the host immune response. An interest in the role of ASA as adjuvant therapy for leprosy rose since the 1980s. Prostaglandins are involved in the regulation of the normal immune response and previous studies examined the potential effects of prostaglandins on leprosy, hypothesizing that the inhibition of prostaglandins synthesis by n-3 fatty acids may cause immunostimulation, promoting the resolution of leprosy lesions [99]. ASA in leprosy is also likely to act by the induction of ASA-triggered lipoxins, a similar effect to that observed with *M. tuberculosis* [100]. In a double-blind controlled trial, 34 cases of type-2 lepra reaction were divided in groups of treatment with colchicine or ASA (1.8 g/day) in addition to standard therapies. Both drugs examined were found equally effective in mild degree reaction [101]. In a more recent study, ASA (2.8 g/day) was added to clofazimine in six patients with erythema nodosum leprosum. The treatment showed favorable results with almost complete disappearance of neutrophils and marked reduction in symptoms. No recurrence was observed at 6 months follow-up [102].

### 3.4. Aspirin and Fungal Infections

The existence of aspirin-sensitive 3-hydroxy fatty acids (i.e., 3-OH oxylipins) in yeasts was first reported in the early 1990s. These oxidized fatty acids are produced by mitochondria especially during sexual stages and act as strong proinflammatory lipid mediators. ASA inhibits mitochondria and thus 3-OH oxylipin production. Yeasts (e.g., *Cryptococcus* spp.) with a mitochondrion-dependent strict aerobic metabolism and/or in sexual developmental stages are indeed more susceptible to ASA [103,104,105].

With regard to *Cryptococcus* spp., ASA has been evaluated as an alternative drug to control cell growth. An in vitro study on ten *Cryptococcus* strains demonstrated that ASA acts in synergy with fluconazole and amphotericin B in enhancing macrophage-mediated phagocytosis, killing cryptococcal cells and inhibiting cellular growth [106].

The interaction mechanisms between ASA and *Candida* spp. have also been addressed. In a reconstituted human tissue model, ASA demonstrated the ability to reduce fungal pathogenesis by inhibiting lipolytic activities. Lipases are in fact part of the virulence factors of *Candida* and catalyze a number of reactions, being also possibly involved in fungal nutrition and adhesion to host cells and tissues and the initiation of inflammatory processes by interaction with the immune cells [107].

Other ASA-mediated antifungal pathways, e.g., effects on neutrophil migration and cytokine production, have not been entirely confirmed [108].

A recent study indicated that verapamil combined with fluconazole could suppress biofilms of *C. albicans* by blocking calcium channels on the cell membrane and disrupting the calcium balance, eventually leading to fungal death. On this basis, *C. albicans* strains were treated in vitro with a combination of caspofungin plus ASA, confirming that ASA can be a *sensitizer* for caspofungin, reducing its MIC50 and inhibiting fungal growth under either planktonic or biofilm conditions [22].

### 3.5. Aspirin and Parasitic Infections

The role of ASA in parasitic infections has not been thoroughly investigated, except for Chagas disease. Chagas disease is a zoonosis caused by *Trypanosoma cruzi*, a protozoan flagellate endemic in Central and South America, as well as in the Southern United States [109]. Transmission may occur through three main mechanisms: vectorial (via blood-sucking triatomine insect bite), congenital and through blood and blood products [109]. Oral transmission is also possible, through the ingestion of food contaminated by infected triatomine or their feces [110]. Recently, the effect of ASA treatment on *T. cruzi* infection by the oral route was investigated in murine models. ASA treatment before *T. cruzi* infection caused gastric mucosal injury in mice and facilitated the invasion of *T. cruzi* trypomastigotes [111].

From a clinical point of view, in Chagas disease, we can distinguish three phases: acute, indeterminate and chronic. The chronic phase of the disease could develop 10–30 years after acute infection. It involves the cardiovascular system, the digestive system or both [109]. Several studies show that ASA could alter the natural course of Chagas disease.

The effect of ASA in reducing the parasitemia in the acute phase is quite well studied, but the findings are contradictory [112,113,114,115]. In a murine model of chronic Chagas disease, ASA decreased the amount of cardiac inflammatory infiltrates, showing a vascular-protective role in the endothelium [116]. The chronic digestive disease consists of megaesophagus or megacolon or both [109]. The main pathogenetic mechanism involved in the chronic digestive disease is the denervation of the enteric nervous system [109,117]. Administration of ASA was found to be neuro-protective in mice models [118,119], leading to an increase in the number of nitrergic neurons and preventing hypertrophy of the esophagus [118,119] and the colon [113]. Recently, De Souza et al. found that ASA does not have a neuro-protective function in mice infected by *T. cruzi*, contradicting the results of previous studies [114]. Only two drugs are approved for the treatment of Chagas disease: nifurtimox and benznidazole [109]. Few studies were conducted about the effectiveness of ASA in combination with standard therapy. In 2009, Lopez-Munoz et al., for the first time, described the synergistic effect of ASA with nifurtimox and benznidazole in an in vitro model of *T. cruzi* acute infection [120]—this combination improved the antiparasitic activity of macrophages. Recently, the combination of benznidazole and ASA was proved effective in mice in the acute phase of the disease, preventing the evolution to cardiovascular dysfunction, and in the chronic phase, decreasing typical cardiac lesions of the disease [121].

The effect of ASA in malaria was also investigated. In 1994, Hemmer et al. conducted a prospective randomized study in Germany on 97 patients infected by *P. falciparum*. They demonstrated that ASA (administered with antiparasitic treatment) does not affect the course of the disease in terms of parasitemia, fever clearance or length of hospital stay [122].

Only two substantial studies were conducted on the role of ASA in helminthic infections.

*Schistosoma mansoni* is a trematode that causes gastro-intestinal schistosomiasis, which is endemic in Africa, the Arabian peninsula and South America [123]. Feitosa et al. found that in mice models, ASA administered with menthol and menthone act in the modulation of the inflammatory process during disease (granulomas) and might help during hepatic inflammation and fibrosis control [124].

*Opisthorchis viverrini* is a trematode that causes opisthorchiasis, a parasitic disease endemic in Southeast Asia [125], which is one of the major risk factors for cholangiocarcinoma. Sudsarn et al., in 2015, determined that the administration of low doses of ASA in addition to the antiparasitic therapy (praziquantel) reduced liver fibrosis and reduced/prevented cholangiocarcinoma development in hamsters [126].

### 3.6. Aspirin and Viral Infection

#### 3.6.1. Aspirin and Human Immunodeficiency Virus Infection

Several studies explored the effects of ASA in human immunodeficiency virus (HIV) infection. Many focused on the prevention of cardiovascular events, but some investigated the potential of ASA in reducing inflammation and viral replication.

Antiretroviral therapy (ART) significantly increased life expectancy in people living with HIV (PLWH). This led to an increasing number of non-HIV-related comorbidities and deaths, especially cardiovascular diseases (CVD) [127]. The European AIDS Clinical Society (EACS) recommends the use of ASA only in PLWH with established CVD or with diabetes and elevated underlying CVD risk [128].

In 2002, the impact of some non-steroidal anti-inflammatory molecules on HIV replication was investigated. In particular, o-(acetoxyphenyl)hept-2-ynyl sulfide (APHS), a synthesized aspirin-like molecule, was tested in different stages of infection of human primary cells. Experiments demonstrated that APHS inhibited the replication of several HIV-1 strains at non-toxic concentrations. This effect was explicated before provirus integration into the host genome, probably during reverse transcription [129].

Later, a few studies evaluated anti-inflammatory and anti-platelets effects of ASA in HIV. After a promising pilot study demonstrating an attenuation of platelet and immune activation markers after 1 week of low-dose ASA therapy [130], in 2016, O’Brien et al. conducted a double-blind, randomized, placebo-controlled trial on HIV-infected participants on suppressive ART to compare 12 weeks of ASA versus placebo on soluble markers of coagulation (sCD14, IL-6 and D-dimer), serum level of thromboxane-B2 and endothelial dysfunction. No significant difference was found between the placebo and ASA groups [131].

The potential role of ASA in HIV-related neurocognitive disorders has also been investigated [132]. Since the upregulation of arachidonic acid metabolism in the central nervous system could be potentially involved in HIV-related neurocognitive disorders, one HIV animal study measured the level of acid arachidonic derivatives after a low-dose ASA treatment, finding a significant reduction in some arachidonate derivatives’ (15-epi-lipoxin-4, 8-isoprostane, prostaglandin E2 and leukotriene B4) brain concentrations in rats treated with ASA [132].

As ART therapy can contribute to cardiovascular risk, inducing metabolic changes, endothelial dysfunction and platelet hyperreactivity [133], ASA has been studied to reduce ART-related CVD risk. A prospective, randomized, placebo-controlled, double-blind cross-over study published in 2018 investigated the potential role of ASA in abacavir-induced platelet hyperreactivity. Only a slight decrease in platelet hyperreactivity in patients treated with low-dose ASA was detected. Moreover, 42% of the HIV participants treated with abacavir and ASA showed ASA non-responsiveness [134].

Data on using ASA in HIV remain controversial. A review published in 2019 confirmed the inconsistency of evidence supporting low-dose ASA for primary CVD prevention, but strongly recommended its use for secondary prevention, especially in combination with statins [135]. Recently, one case-control study including 38 patients showed a significant reduction in endothelial dysfunction (increase in brachial artery flow-mediated dilation) in HIV-infected patients aged between 40 and 59 years who received a 6-month treatment with atorvastatin and low-dose ASA (OR 4.37, 95% CI 1.07–17.79) [136].

In conclusion, ASA could be an important side therapy in HIV patients. Its use remains essential in the secondary prevention of CVD, even if it is still unclear if HIV patients with high cardiovascular risk could benefit from ASA for primary prevention and why some patients seem to have an impaired responsiveness to ASA. Furthermore, ASA and similar drugs have shown in vitro antiretroviral properties, but further studies are needed to fully understand their mechanism of action.

#### 3.6.2. Aspirin and SARS-CoV-2 Infection

The global pandemic caused by SARS-CoV-2 has over 260 million confirmed cases and 5.2 million deaths worldwide (November 2021) [137]. The clinical presentation of SARS-CoV-2 infection is variable, ranging from totally asymptomatic cases up to mild, severe or life-threatening manifestations related to the disease named COVID-19. Severe lung and systemic inflammation may develop in COVID-19 patients, with potential to induce respiratory failure, multi-organ dysfunction and finally, death. Although still largely unknown, the mechanisms underlying the most severe clinical manifestations involve hyperinflammation (the so-called cytokine storm) and a prothrombotic status, with relevant platelet activation, microvascular thrombosis and embolization. Therefore, a strong pathophysiological rationale for a potential therapeutic role for ASA in COVID-19 has been advocated because of its anti-inflammatory, antiplatelet aggregation and anticoagulant effects, as well as its modulation of the immune system and possible inhibition of viral replication and/or entry [138,139,140].

Since ASA is largely prescribed worldwide for the primary prevention of cardiovascular diseases, several studies retrospectively analyzed the effects of antiplatelet action of low-dose (81–100 mg/day) ASA in COVID-19. Based on this literature, a total of five systematic reviews with meta-analysis were published [140,141,142,143,144]. The most recent was conducted by Martha et al. and included six studies comprising 13,993 patients [141]. Overall, the meta-analysis agreed in finding that the use of low-dose ASA was significantly associated with a reduced risk of mortality compared with patients not undergoing this therapy. Only Martha’s study distinguished between individuals taking ASA routinely and those receiving low-dose ASA during hospitalization and reported that in both cases, ASA was significantly and independently associated with reduced mortality (pre-infection ASA: RR 0.46, 95% CI 0.35–0.61, *p* < 0.001; in-hospital ASA: RR 0.39, 95% CI 0.16–0.96, *p* < 0.001) [141]. However, the meta-analysis also agreed that a low certainty of evidence for the mortality-reducing effect of low-dose ASA can be assumed, mainly because of the retrospective design of the most included studies, with a possibility of relevant biases. For example, the meta-analysis included studies involving from 5 to more than 40,000 patients: only one study performed a separate meta-analysis after excluding such outliers, not confirming the effect of ASA on mortality [144].

Subsequently, two prospective clinical trials were published between October and November 2021. The first included symptomatic clinically stable outpatients with COVID-19 who were treated with ASA 81 mg/day compared to a placebo; unfortunately, this study was interrupted early, having documented an event rate lower than expected [145]. The second was a large multicentric RCT realized by the RECOVERY Collaborative Group, exploring the effect of 150 mg ASA (daily until discharge) compared to usual care in hospitalized COVID-19 patients, finding that ASA did not reduce neither the risk of invasive mechanical ventilation, nor 28-day mortality (RR 0.96, 95% CI 0.89–1.04; *p* = 0.35); conversely, in the ASA group, a statistically significant increase in major bleeding events was documented (1.6% vs. 1.0%; *p* = 0.003) [146].

In conclusion, regarding the potential effects of ASA in patients with COVID-19, the available literature reports conflicting results with low-level evidence, and the only available RCT has not confirmed the positive role of ASA. It should be noted that all the above studies explored essentially the antithrombotic effect of ASA, since at the low doses administered, the anti-inflammatory effects are limited [147]. Therefore, further RCTs are needed to prove a positive role of ASA in relevant outcomes in COVID-19 patients.

#### 3.6.3. Aspirin and Viral Hepatitis

Several scientific studies support the hypothesis that aberrant induction of COX-2 and prostaglandin cascade upregulation play a significant role in viral-mediated cellular damage. Here, we summarize existing data on ASA and hepatotropic viruses.

Rivas-Estilla et al. evaluated the participation of oxidative stress in the negative regulation of hepatitis C virus (HCV)-RNA induced by ASA, demonstrating that ASA treatment reduced oxidative stress marker generation, an effect assessed by reactive oxygen species (ROS) and protein-oxidized measurements [148]. Another interesting effect of ASA on HCV infection has been characterized by Zhang et al.; they showed that ASA can inhibit HCV viral entry through the modulation of the claudin-1 level, an HCV receptor [149]. Clinically, Yen-Hsiang Liao and coworkers conducted a study enrolling 1991 ASA-treated and 1911 non-ASA-treated patients with propensity score matching. The adjusted HR of hepatocellular carcinoma (HCC) incidence in the ASA users was significantly lower than that in non-ASA users [150]. Moreover, Xiaofei Li and colleagues performed a meta-analysis of seven cohort studies involving 120,945 adult patients with hepatitis B virus (HBV) or HCV infection. Pooled results showed that ASA use was independently associated with a reduced risk of HCC [151]. Some preclinical evidence also supports ASA’s role in liver disease prevention and progression for hepatocellular carcinoma and hepatitis. At the cellular level, it has been characterized that the proinflammatory COX-2 enzyme is overexpressed in activated hepatic stellate cells and inflammatory cancers [152]. At the molecular level, COX-2 expression activates profibrotic and proliferative signaling cascades, including protein kinase 3 and nuclear factor κB pathways, which are inhibited by COX-2 inhibitors such as ASA [153]. In preclinical models, selective COX-2 inhibition reduces liver fibrosis [153,154], portal hypertension and proliferation of liver cancer cells [155]. ASA may also contribute to prevent fibrosis and hepatitis through glycoprotein 1b-mediated inhibition of intrahepatic platelet activation, degranulation and immune cell trafficking [156]. Some retrospective observational studies have recently evaluated ASA-related risks of bleeding in patients with chronic hepatitis B in Korea and Taiwan [157], demonstrating that gastrointestinal bleeding events were not significantly more common among users of ASA as monotherapy than among non-users.

These findings contribute to incorporating ASA into guidelines for hepatocellular carcinoma and hepatitis prevention, although further research is needed to define its potential hazards across the complete spectrum of liver diseases.

#### 3.6.4. Aspirin and Other Viral Infections

ASA’s role in viral infection has been studied over the past few decades.

Several studies have been focused on *Herpesiviridae*, with a beneficial effect observed since the 1960s. ASA was proven in 1962 to significantly reduce HSV recurrency [158].

Another extremely common herpesvirus, human cytomegalovirus (CMV), is believed to lead to atherosclerosis in smooth muscle cells through reactive oxygen species (ROS) generation. ROS production is neutralized by ASA, thus inducing an inhibitory effect on NFkB-mediated gene expression [159]. Later studies proved ASA was able to stop CMV replication in vitro [160].

Not all these discoveries have been transposed into human clinical studies. Some trials have been performed to treat herpetic neuralgia associated with varicella zoster virus (VZV). A mixture of ASA and chloroform or diethyl ether has shown efficacy in the treatment of acute and postherpetic neuralgia [161] through a topical pain reliever effect at the cutaneous nociceptor level [162]. ASA’s mechanism of action was investigated in vitro in more recent studies and a direct effect on VZV foci formation was observed [163].

Besides a therapeutic effect in the course of infection, benefits could also be envisaged in prophylactic terms. Karadi and colleagues in 1998 demonstrated that daily ASA given for months to patients with recurrent facial–oral and genital HSV infection halves the number of days with active infection [164].

The potential of ASA to treat Epstein Barr Virus (EBV) was also investigated. As for other herpesvirus, EBV lytic cycle induction is inhibited by basal NF-kB activity. Liu et al., in 2008, showed that ASA inhibits NF-kB nuclear translocation and induces EBV lytic cycle induction, triggering EBV-positive cell death [165].

The activity of ASA in the NF-kB pathway has also been studied in the context of other viral infections. Human respiratory syncytial virus (RSV) is a major causative agent of respiratory disease and death in young children. Bitko and colleagues showed that ASA inhibited transcription of NFK-B related genes, by reducing cytokine induction by RSV through post-translational depletion of NF-kB cascade [166].

ASA was found in vitro to be highly effective against influenza A H1N1 virus, coxsackievirus (subtype A9), human rhinoviruses A subtype 1 and 2 and human rhinovirus B subtype 14 and 39 [138]. Regarding rhinoviruses, the effect was not associated with a limited viral shedding [167]; rather, it was based on a systemic antibody response, as previously demonstrated by Hsia [168]—indeed, oral ASA induces the production of cytokines such as interferon-gamma and interleukin-2, through acetylation of macrophage cyclooxygenase. A more direct effect appears to be the inhibitory effect of ASA on Singapore influenza virus infections (H1N1, responsible for the 1986 pandemic flu). Huang and colleagues demonstrated in vitro that ASA-induced inhibition occurs through the prevention of the synthesis of two viral proteins (hemo-agglutinin and nucleocapsid) and the suppression of viral spread, even though data were reported using high concentrations of ASA, difficult to reach in clinical practice [169].

It is important to remember that the Food and Drug Administration and the Centers for Disease Control and Prevention recommend that ASA should not be used to treat acute febrile viral illness in children and teenager less than 19 years old, since its use it is associated with Reye syndrome, an acute, noninflammatory encephalopathy and hepatotoxicity that follows an acute viral illness. Since 1986, when the FDA required that ASA labels state that children and teenagers should not use the product, a drop of about 500% in Reye syndrome cases has been observed, and today, Reye syndrome is rare [170].

## 4. Conclusions

This review provided a comprehensive overview of the current knowledge on the potential of role of ASA in infectious diseases. What emerged is that ASA is a thoroughly studied molecule in different aspects of its effects on microorganisms and their associated infections. Far from being a panacea, different, sometimes surprising and even paradoxical effects of ASA were documented, mainly through non-clinical research. In addition, the interpretation of human studies should take into account the phenomenon of “aspirin resistance” (10% of patients) [171]. In light of a continuous flow of evidence of ASA’s effects in this field of human health, this review may help clinicians and researchers further explore the potential of ASA, as it is likely that some of ASA’s mechanisms of action are yet to be discovered. We believe that the combined anti-inflammatory and anti-infective properties of this drug will be further explored in order to provide indications for its use as a companion drug during clinical infectious diseases.

## Figures and Tables

**Table 1:** Summary of the most relevant results of the literature review (see the main text for further details and specific references).

ASA’s Target		Main Results of ASA Exposure
Platelets		ASA limited platelet activation and inflammatory factor release induced by *Staphylococcus aureus* and *Enterococcus faecalis*
Biofilm		Good results against *Candida albicans* biofilm ASA reduced streptococcal heart valves adhesion
Bacteria	*General considerations*	Antibacterial effects (Gram positive > Gram negative) Anti-chlamydial activity at high doses Increasing/decreasing OMPs, efflux pumps and up-regulating antibiotic targets ASA partially or totally reverted resistance to colistin induced by CCCP in *Escherichia coli* and *Klebsiella pneumoniae*
	*Endotoxin*	Conflicting results Recent studies in human volunteers treated with ASA and exposed to LPS showed a proinflammatory phenotype (↑ TNF-α, ↑ IL-6, ↑ IL-8 and ↓ IL-10)
	*BSI/sepsis*	Retrospective study: low-dose ASA associated with reduced mortality in patients with *S. aureus* BSI, but not *E. coli* BSI ANTISEPSIS trial: no differences among ASA exposed vs. controls (only 5 deaths due to *S. aureus*) Likely differences according to etiologic agent tested
	*Endocarditis*	Meta-analysis: significantly reduced embolism, no differences in bleeding, trend toward higher mortality in ASA-treated patients with IE
	*Gastroenteritis and liver abscess*	Controversial results for gastroenteritis ASA reduced the risk of pyogenic liver abscess (↑ phagocytosis)
	*PJI*	Patients with PJI and ASA exposure had a benefit on infection resolution
	*Pneumonia*	ASA reduced cardiovascular events after pneumonia Observational studies in favor of a reduced mortality when ASA + macrolide are administered in patients with pneumonia
	*Mycobacterial diseases*	Reduced risk of new cerebral infarction in patients with tuberculous meningitis In diabetic patients with pulmonary TB and ASA exposure → improvement of clinical signs and symptoms and cavitary lesions, reduced inflammatory markers and higher rate of sputum-negative conversion
Fungi		↑ macrophage-mediated phagocytosis (*Cryptococcus*) ↓ lipolytic activities of *Candida* (↓ pathogenesis) ↓ *C. albicans* MIC50 to caspofungin (sensitizer)
Parasites		Heterogeneous and weak results Chagas disease → the addition of ASA to nifurtimox and benznidazole improved the antiparasitic activity of macrophages
Viruses	*HIV*	Results too weak to support ASA use in primary prophylaxis
	*SARS-CoV-2*	The only existing trial did not show reduction in IMV or mortality in ASA group
	*Viral hepatitis*	ASA reduced HCC risk in HBV and HCV patients ASA contributed to fibrosis prevention in patients with viral hepatitis
	*Others*	Good results in inhibiting HSV, CMV, VZV, EBV

ASA: acetylsalicylic acid; BSI: bloodstream infection; CCCP: carbonyl cyanide m-chlorophenylhydrazone; CMV: cytomegalovirus; EBV: Epstein Barr virus; HBV: hepatitis B virus; HCC: hepatocellular carcinoma; HCV: hepatitis C virus; HIV: human immunodeficiency virus; HSV: herpes simplex virus; IE: infective endocarditis; IMV: invasive mechanical ventilation; LPS: lipopolysaccharide; OMPs: outer membrane proteins; PJI: prosthetic joint infection; TB: tuberculosis; VZV: varicella-zoster virus.

## Data Availability

Not applicable.
